# Prognostic significance of metallothionein expression in renal cell carcinoma

**DOI:** 10.1186/1477-7819-3-5

**Published:** 2005-01-17

**Authors:** Dionisios Mitropoulos, Aspasia Kyroudi-Voulgari, Stamatis Theocharis, Efraim Serafetinides, Epaminondas Moraitis, Anastasios Zervas, Christos Kittas

**Affiliations:** 1 Department of Urology, University of Athens, Medical School, Athens, Greece; 2 Department of Histology & Embryology, University of Athens, Medical School, Athens, Greece; 3 Department of Forensic Medicine & Toxicology, University of Athens, Medical School, Athens, Greece

## Abstract

**Background:**

Metallothionein (MT) protein expression deficiency has been implicated in carcinogenesis while MT over expression in tumors is indicative of tumor resistance to anti-cancer treatment. The purpose of the study was to examine the expression of MT expression in human renal cell carcinoma (RCC) and to correlate MT positivity, the pattern and extent of MT expression with tumor histologic cell type and nuclear grade, pathologic stage and patients' survival.

**Patients and methods:**

The immunohistochemical expression of MT was determined in 43 formalin-fixed and paraffin-embedded RCC specimens, using a mouse monoclonal antibody that reacts with both human MT-I and MT-II. Correlation was sought between immunohistochemical (MT positivity, intensity and extension of staining) and clinico-pathological data (histological cell type, tumor nuclear grade, pathologic stage and patients' survival).

**Results:**

Positive MT staining was present in 21 cases (49%), being mild/moderate and intense in 8 and 13 cases, respectively. The pattern was cytoplasmic in 7 cases and was both cytoplasmic and nuclear in 14 cases. MT expression in a percentage of up to 25% of tumor cells (negative MT staining included) was observed in 31 cases, in a percentage 25–50% of tumor cells in 7 cases, and in a percentage of 50–75% of tumor cells in 5 cases. There was no significant correlation of MT intensity of staining to histological type, stage and patients' survival, while it was inversely correlated to higher tumor nuclear grade. MT extent of staining did not correlate with histological type, nuclear grade, and pathologic stage while a statistically significant association was found with patients' survival.

**Conclusions:**

The inverse correlation between MT staining intensity and tumor nuclear grade in RCC suggests a role of MT in tumor differentiation process. Since extent of MT expression is inversely correlated with survival it may be possibly used as a clinical prognostic parameter.

## Background

Metallothioneins (MTs) were firstly discovered by Margoses and Valle in 1957 [1] as cadmium (Cd) binding proteins. Later, Piscator [2] documented a marked increase of MT in Cd exposed rabbits, as a metal detoxification mechanism. MTs are a family of heavy metal binding proteins with a large degree of sequence homology that have been described in most vertebrate and invertebrate species. They are single-chain proteins, with molecular weight of approximately 6000 Da, characterized by a very high proportion of cysteine residues (30%), resulting in several high affinity Cd and/or zinc (Zn) binding sites [3]. There are two major isoforms, referred to as MT-I and MT-II [4], resolvable through ion exchange chromatography, that have closely related but distinct amino acid sequences and are distributed in most adult mammalian tissues. Recently, a further charge-separable MT isoform (MT-0) [4], and genes for two MT isoforms with restricted tissue distribution MT-III (brain neurons) [5,6] and MT-IV (stratified epithelia) [7] have been described. The potential for wider tissue distribution of MT-III was suggested by recent studies demonstrating the presence of MT-III mRNA and protein in the adult and developing human kidney [8,9].

MT-I and MT-II isoforms are usually expressed in low levels, but are inducible by a variety of metal ions, hormones, inflammatory cytokines and xenobiotics [10-12]. Induction of MTs is important in detoxification and metal ion homeostasis [9], in protection against reactive oxygen species [10] and in tissue regeneration [13-15]. MT expression deficiency implicated in carcinogenesis [16] and possible relation of MT over expression and resistance of tumors to anti-cancer therapy [17] has provided evidence of the importance of MT expression in cancer. MT over expression, detected immunohistochemically, has been described in a variety of human tumors, in relation to different stages of tumor development and progression [18]. The involvement of MT and Zn, in processes such as p53 gene activation and protein structure has been referred [16,19]. There is evidence that some human tumors contain high levels of MT, nevertheless, the importance of MT expression in carcinogenic evolution and in patients' survival is not yet fully understood.

In organs such as kidney, colon and liver, normally implicated in metal ions homeostasis, MT protein is apparently expressed. It would be of great scientific importance to elucidate the pattern of MT expression in tumors developed from these organs, since it could not only delineate the role of MT in carcinogenic transformation but could also provide prognostic information for patients' outcome. The aim of the present study was to examine the expression of MT in human renal cell carcinoma (RCC) and to correlate the MT positivity, the pattern and extent of MT expression with tumor histological cell type and nuclear grade, pathologic stage and patients' survival.

## Patients and methods

Forty three consecutive patients, 31 men and 12 women, who underwent nephrectomy for RCC comprised the group of our study. Their age ranged from 33 to 85 years (mean age 59.6 ± 11.1). Tumors were histologically classified as clear cell type in 32 cases, papillary type in 2 cases, chromophobe cell type in 4 cases and sarcomatoid type in 5 cases. The 8, 2, 16, 6 and 9 tumors were pathologically staged as T1 or T2N0M0, T2N+M0, T3N0M0, T3N+M0 and T4N+M+, respectively as per the TNM classification [20]. The grade of nuclear atypia according to Fuhrman Grading system [21] was: grade I in 11 cases, grade II in 15 cases, grade III in 6 cases, and grade IV in 11 cases. The patients were followed up from 2 up to 144 months (5 lost in follow up), mean 65.4 months, median 39 months.

### Immunohistochemistry

Sections of 5 μm thickness were deparaffinized in xylene and rehydrated in graded alcohol series. To remove the endogenous peroxidase activity, sections were treated with freshly prepared 0.3% (v/v) hydrogen peroxide in methanol in dark, for 30 min, at room temperature. Non-specific antibody binding was then blocked using normal rabbit serum (Dakopatts, Glostrup, Denmark) diluted 1:5 in phosphate buffered saline (PBS), for 20 minute. A mouse (IgG1k) monoclonal antibody that reacts with both human MT-I and -II isoforms (Zymed, San Francisco, California, USA) was used in this study. The sections were then incubated for 1 hour, at room temperature, with the primary antibody diluted 1:50 in PBS. After three washes with PBS, sections were incubated for 30 minute at room temperature with rabbit, peroxidase conjugated, anti-mouse serum (Dakopatts) diluted 1:200 in PBS and rinsed three times with PBS. Sections were then incubated with swine, peroxidase conjugated, anti-rabbit serum (Dakopatts) diluted 1:100 in PBS and rinsed three more times with PBS. The resultant immune peroxidase complexes were developed in 0.5% (v/v) 3,3'-diaminobenzidine hydrochloride (DAB; Sigma, Saint Louis, MO, USA) in PBS containing 0.03% (v/v) hydrogen peroxide. Sections were counterstained with Harris' hematoxylin and mounted in gelatin (Sigma). Control slides included in MT immunostaining procedure consisted of specific tissues previously shown to express MT (lung cancer) as positive controls, whereas the primary antibody was replaced by PBS in the case of negative controls.

### Scoring system

The stained sections were independently assessed by the pathologists (A.K., S.T., E.M.) without prior knowledge of the clinical data as previously described [22]. Specimens were considered as "positive" for MT when more than 5% of tumor cells within the section were positively stained. The intensity of staining was graded as mild (+), moderate (++), and intense (+++). To further evaluate the importance of staining extent, cases were stratified into 3 groups according to the percentage of cells staining positive for MT: group A, 0–25%; group B, 26–50%; and group C, >50% of MT positive cells. The pattern of MT staining was also characterized as cytoplasmic only, nuclear only, and both cytoplasmic and nuclear.

### Statistical analysis

Correlation between immunohistochemical data (MT intensity and extent of staining) and clinicopathological data (histologic cell type, nuclear grade, pathologic stage) was assessed using the Chi-square test. The association of intensity and extent of MT immunohistochemical staining with survival was determined by comparing Kaplan-Meier survival curves constructed for different patient groups, and were compared using the log-rank test.

## Results

**Figure 1:**
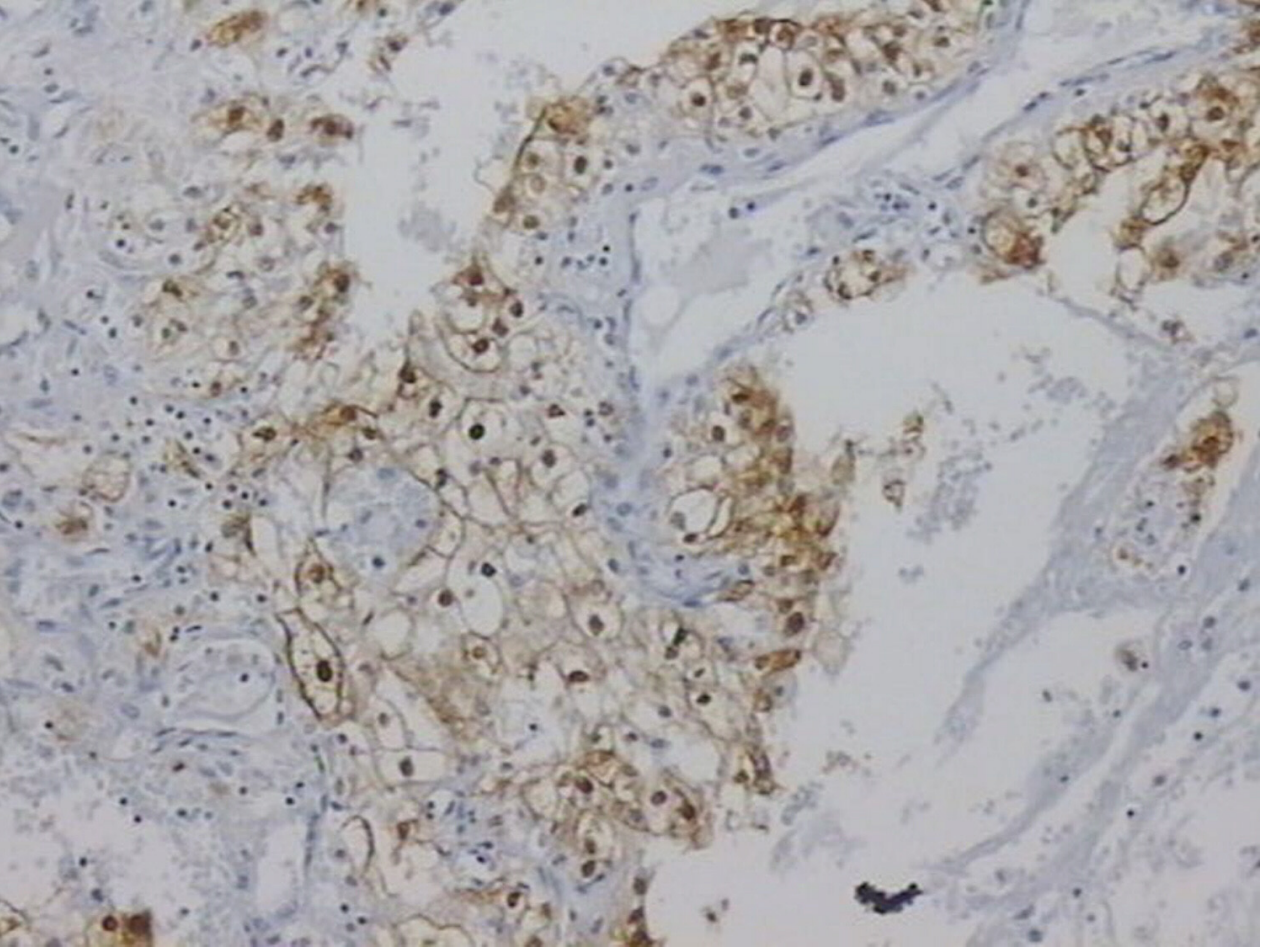
Detection of MT expression by immunohistochemistry. Intense membranous, cytoplasmic and nuclear staining in a case of clear cell type RCC (x330).

Tubular cells but not glomeruli and interstitial cells of normal autologous renal tissue stained positive (both in the nucleus and the cytoplasm) for MT, although the intensity and extent varied significantly. Positive MT expression was prominent in 21 out of 43 cases (49%), while 22 out of 43 ones (51%) were MT negative. As far as the intensity of staining is concerned, low/moderate intensity was observed in 8 cases, while intense staining was evident in 13 cases. The pattern of positive MT immunostaining observed was either cytoplasmic (7 out of 21 cases, 33.3%) or cytoplasmic and nuclear (14 out of 21 cases, 66.6%). In certain cases of clear cell RCC membranous staining was also observed. Nuclear pattern of staining only, was not observed in any of the RCC cases examined (Figure 1). The extent of MT expression in a percentage of up to 25% of tumor cells (negative MT staining included) was observed in 31 out of 43 cases, in a percentage 25 up to 50% of tumor cells in 7 cases, and in a percentage of 50–75% of tumor cells in 5 cases.

**Figure 2:**
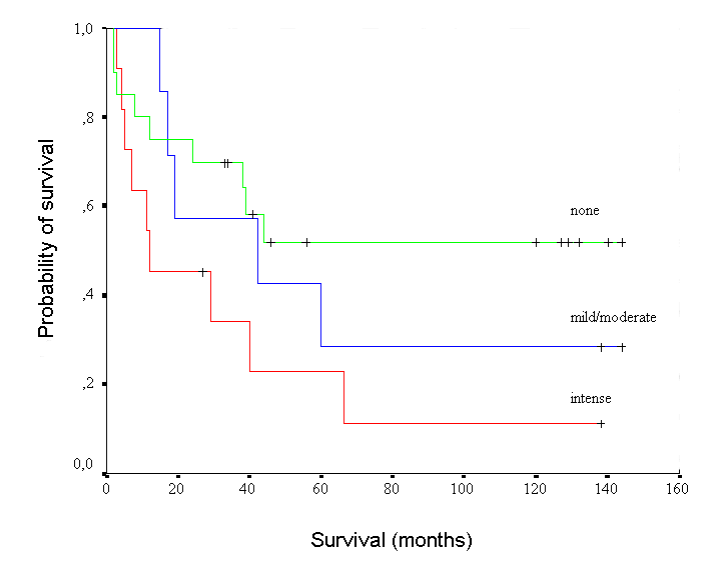
Cancer-specific survival of patients according to intensity of staining for MT (no staining, green line; mild/moderate staining, blue line; intense staining, red line). No statistically significant difference was detected (p = 0.09).

There was no significant difference between MT intensity of staining and the histological types of RCC cases examined (χ^2 ^= 5.61, p = 0.46). No statistically significant differences were also observed between MT intensity of staining and stage (χ^2 ^= 9.24, p = 0.32), or patients' survival (log rank test: 4.75, p = 0.09) in the cases of RCC examined (Figure 2). Statistically significant correlation was found between MT intensity of staining and histological grade, where higher intensity of staining was found in cases presenting high histological grade (χ^2 ^= 13.63, p = 0.03). However, no such correlation was found in the case of clear cell RCC (χ^2 ^= 4.75, p = 0.57).

**Figure 3:**
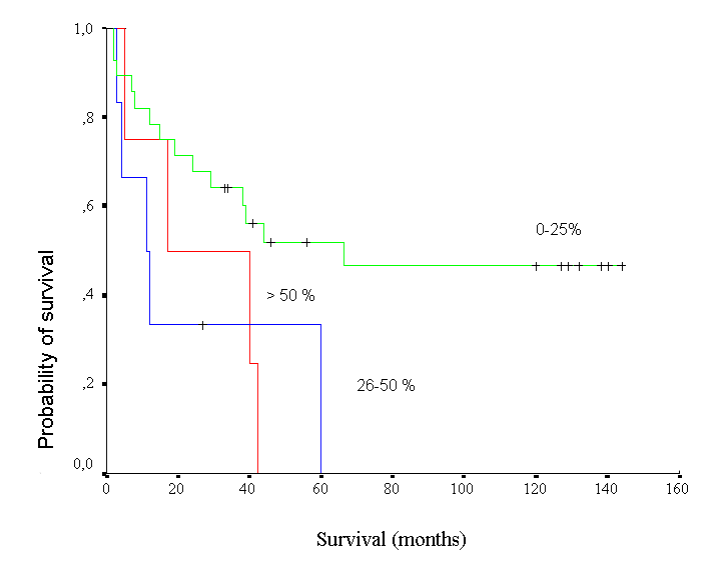
Survival curves of patients according to MT extent of staining (0–25% of cells, green line; 26–50% of cells, blue line; >50% of cells, red line). Statistically significant inverse correlation was found between extent of staining and patients' survival (p = 0.037).

Non statistical correlation was found among MT extent of staining and histological types (χ^2 ^= 2.54, p = 0.86), stage (χ^2 ^= 7.12, p = 0.52) and grade (χ^2 ^= 6.24, p = 0.39) of RCC cases examined. Statistically significant inverse correlation was found between MT extent of staining and patients' survival (log rank test: 6.59, p = 0.037) (Figure 3). Again, this was not observed in case all other histological types but clear cell RCC were excluded (log rank test 3.36, p = 0.186).

## Discussion

MT expression has been observed in different types of human tumors [18,22], including neoplasias of the urogenital tract [23-28]. However, the biological mechanisms underlying MT over expression in tumors, or the consequences of this over expression are not currently well understood. MT is highly expressed in fetal life [10]; the reappearance of this fetal characteristic in tumors suggests its participation in cellular growth and differentiation [16,18,19].

Developing and adult kidneys consistently express MT-I and MT-II mRNA [29] and the corresponding protein [28], while the MT-III isoform is also expressed in developing renal tissue, adult proximal tubule and renal cell carcinoma cell lines [8,9]. The MT-0 isoform is absent in adult kidneys but it can be found in nonneoplastic tissue from renal and transitional cell carcinoma [30]. The isoform specific expression of MT in RCC has not been so far investigated. Using a monoclonal antibody that reacts to both MT-I and MT-II, we demonstrated positive immunoreaction in 49% of our RCC cases, a percentage close to the 55.7% reported by Tüzel *et al *[24], using the same antibody. Zhang and Takenaka [24] used another commercially available monoclonal antibody with unspecified specificity towards different MT isoforms and found positive immunoreaction for the MT protein in 33% of their cases, while such report is not included in the study of Izawa *et al *[23] who used a polyclonal antibody prepared by their own laboratory.

The cytoplasm and nucleus of normal and malignant cells may both express MT but there is no conclusive data on the functional significance of their subcellular distribution. The majority of the cases in our study expressed MT in both cytoplasm and nucleus, the expression being cytoplasmic only in one third of the cases. Analogous pattern has been reported in other studies [23-25] while the membranous staining observed in some of our cases as well as in two previous series [24,25] could be explained by the nature of clear cell carcinoma. Differential subcellular expression of MT may be related to either cell proliferation or the induction of apoptosis [31,32]. Recently, Kondo *et al *[33] pointed out the importance of subcellular distribution of MT in drug resistant-prostatic cancer cells, in which the nuclear MT expression rather the cytoplasmic counterpart seems to predominantly confer resistance to cisplatin. On the other hand, nuclear pattern of MT immunostaining has already been correlated with cellular response to stress stimuli [34]. The function of MT to protect the cell from apoptosis could be an explanation for MT over expression observed in high grade cases of RCC [24], as MT is not etiologically correlated with the apoptotic process.

In certain tumors MT over expression has been associated with unfavorable prognostic characteristics such as advanced stage and poor differentiation [18]. In our study, MT intensity of staining did not correlate with stage while it showed an inverse correlation with histological grade. Our results are in accordance to those of other investigators [23,25], who also found an inverse relationship between MT immunoreactivity and tumor grade. In contrast, Zhang and Takenaka [24] reported positive associations between MT expression and tumor grade. The inverse relationship between MT immunohistochemical expression and tumor grade may suggest a role of MT in cellular growth and differentiation, and reflect alterations of intracellular processes leading to a gradual decline of Zn storage and to the subsequent decrease in MT expression. Although no association was found between MT staining intensity and survival, the reduced extent of MT expression significantly correlated with prolonged survival as reported elsewhere [25]. The extent of MT expression may offer an additional, prognostic factor in patients suffering from RCC.

MT concentrations might also prove useful in predicting the efficacy of a particular cancer treatment protocols. Several types of transformed cells enriched for MT have been shown to exhibit greater resistance to chemotherapeutic agents [17]. In this context, the thiolate sulfur of the cysteine residues are thought to act as sacrificial scavengers for radicals and alkylyating agents [10]. Thus, MT might serve as a Cu and Zn reservoir, where their supply may negatively affect the growth of tumor cells. MT could also participate in tumorigenesis by sequestering and then donating these essential bivalent cations to proteins of tumor cells in order to meet metabolic requirements [35].

## Conclusions

The current data on the expression of MT in RCC cases examined emphasize the necessity to investigate larger numbers of patients with RCC comparing the staining profile of different MT isoforms with other clinico-pathological parameters and survival status of patients. Currently, it is unknown whether the presence of MT in renal carcinoma cells is related to the induction or inhibition of apoptosis or plays an active role in cell proliferation. Since cytokines may also induce MT expression and immunotherapy is the only, albeit with limited efficacy, available treatment for RCC, the intensity and extent of MT immunostaining should be studied in correlation to the immune status of RCC patients before and after immunotherapy. Thus, it will be possible to elucidate the potential role of MT in renal cell carcinogenesis, as well as its clinical usefulness as a tumor marker and as a tool for selecting patients for adjuvant immunotherapy.
